# Two feedback mechanisms involved in the control of leaf fragment size in leaf-cutting ants

**DOI:** 10.1242/jeb.244246

**Published:** 2023-06-23

**Authors:** Daniela Römer, Rebecca Exl, Flavio Roces

**Affiliations:** Department of Behavioural Physiology and Sociobiology, Biocenter, University of Würzburg, Am Hubland, 97074 Würzburg, Germany

**Keywords:** *Atta sexdens*, Foraging, Herbivory, Mechanosensory hairs, Behavioural plasticity, Cutting geometry

## Abstract

Polymorphic leaf-cutting ants harvest leaf fragments that correlate in size with the workers' body size. When cutting, workers anchor their hind legs on the leaf edge and rotate, removing approximately semicircular fragments. Workers show behavioural plasticity and modify their leg extension while holding onto the leaf edge depending on, for instance, leaf toughness, cutting smaller fragments out of tough leaves. What sensory information workers use to control the cutting trajectory remains unknown. We investigated whether sensory information from both the leg contact with the leaf edge and from head movements underlies fragment size determination. In the laboratory, we recorded *Atta sexdens* workers cutting standardised ^®^Parafilm pseudoleaves of different thickness, and quantified cutting behaviour and body reach, i.e. the distance between the mandible and the anchored hind leg tarsus. Experimentally preventing contact with the leaf edge resulted in smaller fragments, evincing that workers control the cutting trajectory using information from the contact of the hind legs with the leaf edge. However, ants were able to cut fragments even when contact of all six legs with the edge was prevented, indicating the use of additional sensory information. Ablation of mechanosensory hairs at the neck joint alone did not influence fragment size determination, yet simultaneously preventing sensory feedback from both mechanosensory hairs and edge contact led to a loss of control over the cutting trajectory. Leaf-cutting ants, therefore, control their cutting trajectory using sensory information from both the leg contact with the leaf edge and the lateral bending of the head.

## Teaser

**Highlighted Article:** Plasticity in fragment size determination in leaf-cutting ants is controlled by sensory information provided by both the leg contact with the leaf edge and the lateral bending of the head.

## INTRODUCTION

Colonies of social insects collect vast quantities of resources. A leaf-cutting ant colony can harvest up to 300 kg of fresh plant matter per year (Wirth et al., 2003), comparable to the yield of a medium-size herbivore. The organised foraging pattern of a colony relies on thousands of individual decisions that are assumed to maximise the colony's resource intake. Optimal foraging theory predicts that animals foraging individually should maximise their resource intake (MacArthur and Pianka, 1966). For central place foragers like social insects, which do not collect the food for themselves but transport it back to their colony, evolution appears to have shaped foraging decisions to maximise colony-wide gains even at the cost of individual gains (reviewed in Roces and Bollazzi, 2009). For example, both honeybees and nectar-feeding ants return to the nest with only partially filled crops when nectar flow rate or nectar quality is low (Josens et al., 1998; Núñez, 1982). At the beginning of a foraging bout, grass-cutting ants cut smaller fragments and return faster to the nest for nestmate recruitment, compared with workers from an established foraging column (Bollazzi and Roces, 2011). Although such behaviour leads to an initial suboptimal resource intake for individuals, it was argued that it increases total resource intake at the colony level, because a shortened foraging time enables faster information transfer about discovered food sources.

Based on optimality arguments, foraging social insects should have evolved mechanisms to determine load sizes; for example, nectar crop loads or the size of the prey/seed collected. One simple mechanism involved in load size determination is size matching (Reyes-López and Fernández-Haeger, 2001). Some seed-harvester ants show a positive correlation between forager size and the size of the seed they harvest (Davidson, 1977). The same is found in leaf-cutting ants (Wetterer, 1990), which collect fresh pieces of vegetation, not as food for themselves, but to farm a symbiotic fungus as the colony's food source.

Size matching in leaf-cutting ants results from their seemingly geometric mode of fragment size determination. A worker anchors itself to the leaf edge with its hind leg tarsi, starts cutting, and rotates around the points of contact (example 1 in Movie 1), severing fragments that resemble semicircles (Lutz, 1929; Weber, 1972). The observation of roughly semicircular fragments led to the hypothesis that workers use a simple behavioural rule: leaf fragment size depends on the ant's ‘reach’ (Weber, 1972), irrespective of physical features of the leaves, or the forager's internal state. As leaf-cutting ants are highly polymorphic, with foragers ranging from 1 to 30 mg (Burd, 1995; Roces, 1990), larger ants have a larger reach between their mandibles and their hind leg tarsi (van Breda and Stradling, 1994; Wetterer, 1990), resulting in the cutting of larger fragments.

However, leaf-cutting ants were shown to modify fragment sizes depending on several factors. Leaf physical features such as thickness have a negative effect (Cherrett, 1972; Roces and Hölldobler, 1994; Rudolph and Loudon, 1986). *Acromyrmex lundii* foragers cut smaller fragments at a near food source compared with one farther from the nest (Roces, 1990). Workers also cut smaller fragments as a trade-off for faster recruitment, when confronted with high-quality food sources, or when harvesting deprived (Roces and Hölldobler, 1994; Roces and Núñez, 1993). Foraging is energetically expensive, both while walking (Lighton et al., 1987) and while cutting a fragment, the latter being almost as expensive as insect flight (Ellington, 1991; Roces and Lighton, 1995). Therefore, control of the cutting trajectory and the resulting determination of leaf fragment size should be flexible, presumably to maximise leaf transport rate for the colony, even at the expense of individual performance (Roces and Bollazzi, 2009).

Flexibility in load-size determination requires peripheral control to adjust or modify the cutting trajectory. In mechanistic terms, it is unclear what kind of sensory information workers may use to control and modify the cutting trajectory depending on the factors mentioned above. While the largest possible fragment size may be simply determined by the ant's maximal reach, which is limited because of the leg anchoring while rotating, several mechanisms may be involved in the control of changes in cutting trajectory. (1) Ants may alter their effective reach, i.e. the span of their body from their hind leg tarsi to their mandibles, by flexing their legs while rotating. (2) They may modify the position of their legs during cutting and therefore change the anchoring points. (3) They may use leg contact with the leaf edge, usually by the hind legs, as a kinematic anchor. Both hind legs appear to be consecutively used as pivot points, i.e. first the hind leg on the ant's side where the cut begins, then the other hind leg for roughly the second half of the cut. (4) They may alter the angle between the head and body axis, to cut with variable angles independent of the extension of their legs. Whatever the sensory mechanisms involved, cutting radius does not appear to be simply controlled by the position or extension of the hind legs (van Breda and Stradling, 1994). A study suggested that feedback information provided by changes in head position may be involved in the control of fragment size determination, irrespective of leg extension (van Breda and Stradling, 1994).

**Fig. 1.**
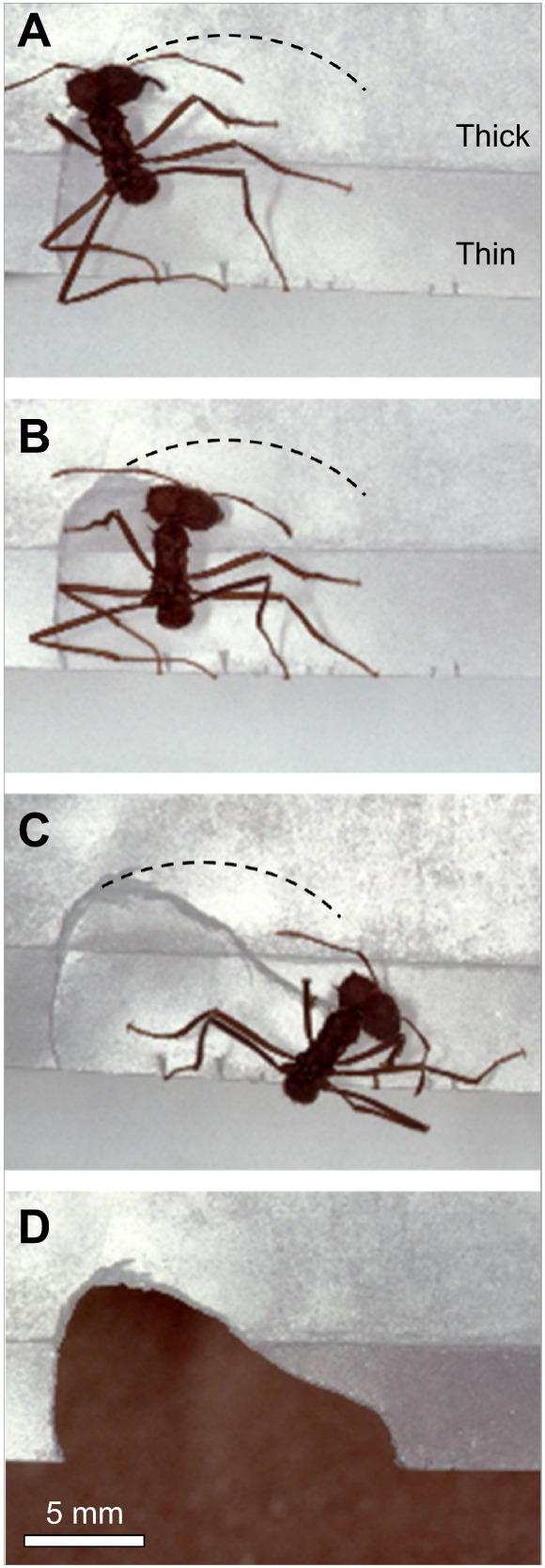
**A forager of *Atta sexdens* cutting a ^®^Parafilm pseudoleaf.** The pseudoleaf was composed of a thin leaf margin (one layer of ^®^Parafilm) and a thick leaf body (three layers of ^®^Parafilm). The dashed line indicates the expected cutting path. Photo credit: H. Heilmann.

Leaf-cutting ants do not simply rotate with a fixed motor pattern around a pivot, as demonstrated with a simple experiment offering a ^®^Parafilm pseudoleaf as standardised and highly acceptable harvesting material (the method of using scented ^®^Parafilm pseudoleaves was previously established by us; Roces, 1990). We prepared a scented pseudoleaf of variable thickness by taking a single ^®^Parafilm sheet of 5×5 cm, and adding a 4×4 cm double-layered piece in its middle, resulting in a ‘composed’ pseudoleaf with a thin margin of 5 mm all around the piece, and a thicker (three layers) inner leaf body (Fig. 1A). Wherever the ant started, it cut first through the thin margin, then the thick part, and finally through the thin margin again. Flexibility in cutting behaviour was evident: the ant started with a cutting trajectory describing an ample arc as expected for the cutting of thin leaves, but altered the trajectory with a smaller radius for the thicker leaf body (Fig. 1B). Thus, ants did not simply pivot while cutting; the cutting trajectory changed dynamically during cutting and did not resemble a semicircle (dashed line in Fig. 1), suggesting that fragment-size determination depends on an unknown force-based mechanism and not on a purely geometric mode. Particularly interesting is the point at which the ant again reached the thin part of the pseudoleaf: instead of cutting straight ahead to finish the cut (Fig. 1C), it followed a completely unexpected convex trajectory and severed the fragment after a final turn (Fig. 1D). What information do ants use to actively alter their cutting trajectory?

The work presented here investigated the sensory information leaf-cutting ants use to control their cutting trajectory. This information underlies their flexible fragment size determination, which is crucial for efficient foraging. We examined the putative involvement of two different feedback loops: first, whether information from leg contact with the leaf edge is used to control the cutting trajectory, and second, whether proprioceptors controlling the head position also provide sensory information relevant to the control of fragment size. Foragers of *Atta sexdens* were allowed to cut pseudoleaves made out of either one layer or three layers of ^®^Parafilm, to mimic thin and thick leaves. Cuts were filmed and digitised to measure the ant's reach when anchoring to the leaf edge over the entire cut, as well as cutting speed, total cutting time and size of the resulting fragment. To evaluate the use of sensory information from contact of the hind legs with the leaf edge, we experimentally prevented potential feedback from the edge contact and quantified both reach during the cut and the resulting fragment sizes. Ablation of episternal mechanosensory hairs at the neck joint allowed us to investigate the involvement of sensory information from the head position in the cutting behaviour of ants.

## MATERIALS AND METHODS

### Study animals

All experiments were performed with three mature colonies of *Atta sexdens* (Linnaeus 1758) collected as founding colonies in Botucatu, Brazil, and maintained at the University of Würzburg, Germany. At the time of the experiments, colonies were either 4 or 5 years old, and consisted of four plastic boxes (19×19×9 cm) filled with fungus gardens, a foraging arena and a waste chamber. Colonies were supplied with fresh leaves (*Rubus fruticosus* and *Ligustrum vulgare*), water and honey solution *ad libitum*.

### Cutting material

We used standardised ‘pseudoleaves’ (5×5 cm squares, based on Roces, 1990) made out of ^®^Parafilm (^®^Parafilm ‘M’ Laboratory film, American National Can, Chicago, IL, USA), which are readily accepted as harvesting material by leaf-cutting ants, to prevent possible influences of leaf veins on cutting behaviour, and, therefore, leaf fragment size. In addition, the laminar thickness of the pseudoleaf could reliably be changed. We used either thin pseudoleaves (thickness: 0.13 mm) made out of one-layer ^®^Parafilm, or thick pseudoleaves (thickness 0.38 mm), made out of three layers that stuck together after having been slightly pressed. To increase acceptance, leaves were scented by either placing them in a closed box with a small Petri dish containing rose oil (Rosenduftöl, Büchener Original, KERMA GmbH, Hainichen, Germany) for 24 h, or slightly smearing them with crushed bramble leaves. Most ants took a firm stance on the pseudoleaf by attaching their leg claws to the surface and edge; only on a very few occasions were workers observed to slightly slide with the legs not anchored to the edge, yet their cutting trajectory was not disrupted.

### Experimental procedure

At the start of an experiment, a pseudoleaf was placed in the foraging arena of an ant colony. When an ant started to cut, the leaf was picked up with tweezers, all ants except the cutting forager were carefully removed, and the leaf was then positioned under a camera (Panasonic F15 High Sensitivity, resolution 756×581 pixels). One point of the leaf was pinned on a pedestal, with the rest of the leaf freely suspended horizontally. This ensured that the forager kept contact with the leaf edge during the entire cut and did not step off the leaf with any legs. Each cut was recorded until the ant had completely severed a leaf fragment. Afterwards, we measured: ant body mass and fragment mass to the nearest 0.1 mg, cutting time (s) and average cutting speed (mm s^−1^, length of the cut divided by the total cutting time from the beginning to the end of the cut). Leaf fragment area (mm²) was extracted using ImageJ software (version 1.44, National Institutes of Health, Bethesda, MD, USA).

### Leaf thickness and fragment size determination

**Fig. 2.**
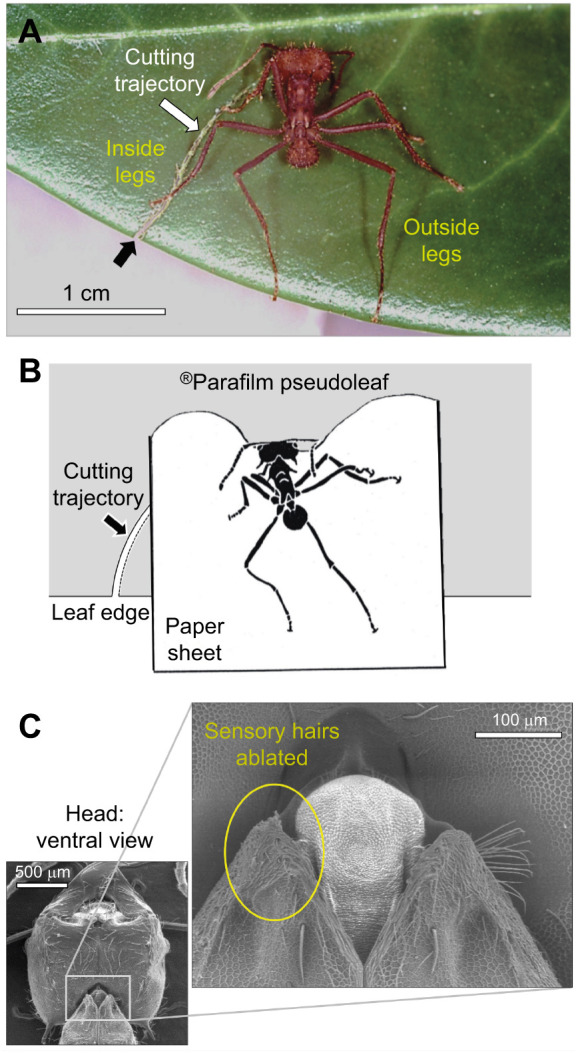
**Methodology.** (A) The definition of ‘inside’ and ‘outside’ legs is based on the initiation of the cutting trajectory. (B) Illustration of the experimental manipulation in which a sheet of paper was inserted between the pseudoleaf and the legs while the ant continued cutting. Ant drawing by Malu Obermayer, with permission. (C) Scanning electron microscope picture of the mechanosensory hair fields on the ventral episternal neck joint of a worker. The inset shows their ablation (yellow circle). In the experiments, hairs from both fields were ablated.

To investigate fragment size determination as a function of leaf thickness, workers were offered either thin or thick pseudoleaves as described above. The foraging ant could display its normal cutting behaviour by anchoring to the leaf edge with its hind legs and pivoting around the two points of contact made by both hind legs (see example 1 in Movie 1). We digitized the *x*–*y* coordinates of the leading cutting mandible and the tarsi of both hind legs in freeze video frames every millimetre over the entire cutting trajectory. The calculated distance between the leading mandible (puncturing point into the pseudoleaf) and the tarsus of each hind leg, to the nearest 0.1 mm, was taken as a measure of the ant's reach (Program Unimark 3.6, Rüdiger Voss Services, Tübingen, Germany). Hereafter, we will refer to the three legs positioned within the initiated cutting trajectory, i.e. the side where the ant started to cut, as ‘inside legs’ (Fig. 2A), and the legs on the other side of the ant, positioned outside the cutting trajectory, as ‘outside legs’. The inside hind leg is therefore the leg anchored to the leaf edge when the cutting started.

### Preventing sensory input from leg contact with the leaf edge

The anchoring of the hind legs to the leaf edge may give the ant sensory feedback about the location of the leaf margin. To preclude the use of this information, we prevented contact of legs with the edge under controlled experimental conditions, using the following method. While an ant was engaged in cutting, we carefully introduced a thin paper sheet (3×3 cm, 120 g m^−2^) between the pseudoleaf and the legs of the ant with only minor physical disturbance while the ant kept on cutting (Fig. 2B). The V-shape of the paper sheet was designed to allow undisturbed head and mandible movements during the complete cut, while preventing the contact of all legs with the pseudoleaf, especially the front legs, which are sometimes placed on the side and above the ant's head. The paper sheet was mounted on a custom-made micromanipulator that allowed fine control of the sheet displacement while it was lying directly on the ^®^Parafilm pseudoleaf. We initiated each manipulation from the point at which the ant's body axis was perpendicular to the leaf edge, which was assumed to represent the first half of the cutting trajectory (see example 2 in Movie 1), as this was a clearly defined point and some handling time was initially needed to set up the experimental conditions. As manipulated ants cut with different trajectories thereafter, the start of the manipulations corresponded to variable percentages of the entire length cut in the figures depicting these results. During the manipulation, the paper sheet needed to be slightly rotated to follow the turning of the ant, so that the targeted legs stayed on it. The rotation of the paper sheet did not influence the ants' cutting behaviour, as workers started first to rotate their body axis, and the paper rotation followed to a much lesser extent (Movie 1).

We performed two experimental series offering either thin or thick leaves, and prevented contact with the leaf edge for all six legs so ants lacked sensory information from the leaf edge as soon as the manipulation started. The size of the fragment cut as well as the reach were quantified as described above. Additionally, a series was performed, using thin pseudoleaves, in which only the sensory input provided by the hind leg contact with the leaf edge was precluded. It was hypothesised that smaller fragments should be cut under such conditions, as ants were expected to try to control the cutting trajectory by anchoring their middle legs to the leaf edge, thus displaying a shorter body reach while rotating. For that, a paper square (3×2 cm) was gently inserted, as described above, to only prevent contact of the hind legs during cutting, allowing free contact of middle and front legs with the leaf edge.

To rule out that the experimental manipulations of inserting a paper sheet while cutting could led to a physical, non-specific disturbance of the normal cutting behaviour, we performed the following control experiment (Fig. S1). The rationale was to experimentally prevent contact of only the inside middle and hind legs (with a paper sheet of 1×3 cm) while the ant continued cutting a thin pseudoleaf, i.e. to perform a manipulation whilst allowing the outside legs to be naturally positioned and to get putative sensory information for the control of the cutting trajectory. If inserting the paper sheet does not cause physical disturbance, it should not affect the expected cutting trajectory. We then compared the fragment sizes cut by manipulated and non-manipulated ants. Fragment sizes did not differ between non-manipulated ants and ants cutting the pseudoleaf with two manipulated inside legs, which indicates that inserting the paper sheet did not physically disturb cutting behaviour (Fig. S1, Table S1A,B).

### Preventing sensory input from the neck joint

To demonstrate that sensory information from the head movements helps workers to control their cutting trajectory, we bilaterally ablated a field of mechanosensory hairs situated at the episternal neck joint (Fig. 2C) and quantified the size of the leaf fragments cut by treated ants. Insects are known to sense their head movement using mechanosensory hairs at that joint (Markl, 1963; Preuss and Hengstenberg, 1992; Wittlinger et al., 2007). We created subcolonies composed of foragers with ablated sensory hairs as follows. Foragers in the process of cutting *R. fruticosus* leaves in the foraging arena of the main colony were collected, lightly anaesthetised with CO_2_, and fixed, ventral-side up, under a binocular microscope. The sensory hairs of the two hair plates were shaved off (Fig. 2C, inset) using a thin, broken-off glass capillary mounted on a vibrating (15,000 Hz) piezo crystal. Each treated ant was marked with a dot of white paint (Edding paint marker) and released back into a subcolony, consisting of a foraging arena and two boxes filled with fungus, separated from the main colony by a gate. Approximately 200 treated foragers were present in the subcolony at any time. The use of a small subcolony increased the probability that treated foragers would voluntarily show up in the foraging box in the consecutive days and cut a thin pseudoleaf. Once the forager had cut a fragment, it was not released back into the colony to avoid pseudoreplicates. Cutting behaviour of treated individuals was recorded at least 24 h after hair ablation to ensure a stress-free response. We performed two experimental series to quantify the size of the leaf fragments cut by workers. In the first, workers with bilaterally ablated sensory hairs could cut a fragment while anchoring their hind legs to the leaf edge, as usual. In the second, workers with bilaterally ablated sensory hairs were in addition manipulated with the paper sheet as described above, so as to prevent contact of all six legs with the leaf edge in the final half of their cut.

### Statistical analysis

The relationships of fragment size, cutting speed and cutting time were evaluated via generalized linear models (GLM) in response to ant mass (centred on the mean), pseudoleaf thickness and leaf type (pseudoleaves and natural leaves), and leg treatment, as linear predictors, using R (www.r-project.org, version 3.6.3 with the package multcomp 1.4-20 for pairwise *post hoc* tests between factor levels). Whenever interaction effects were not statistically significant, we removed them to simplify the model. Differences between groups were compared using the Tukey *post hoc* test. To compare the shape of the fragments cut from pseudoleaves with those of natural leaves, we obtained a measure of their circularity by using a slightly modified ‘form factor’ (Ritter and Cooper, 2009). The form factor is the ratio of the area of an object to the area of a circle with the same perimeter as the object, as follows:

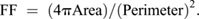
The form factor varies from 0 to 1; the higher the value, the more circular the structure. As fragments cut by ants are roughly semicircular or semi-elliptical, for each fragment we multiplied both the measured fragment area and the cutting length by 2 prior to entering the values in the formula. Therefore, a perfectly semicircular fragment would have a form factor equal to 1. Comparisons of fragment shapes using the form factor, as well as comparisons of the ant reach while cutting, were done using either a *t*-test or a Mann–Whitney *U*-test, depending on normality and equal variance of the data (Sigmaplot 11.0, Systat Software GmbH).

Raw data for Figs 3–9 and Figs S1–S4 are given in Table S5.

**Fig. 3.**
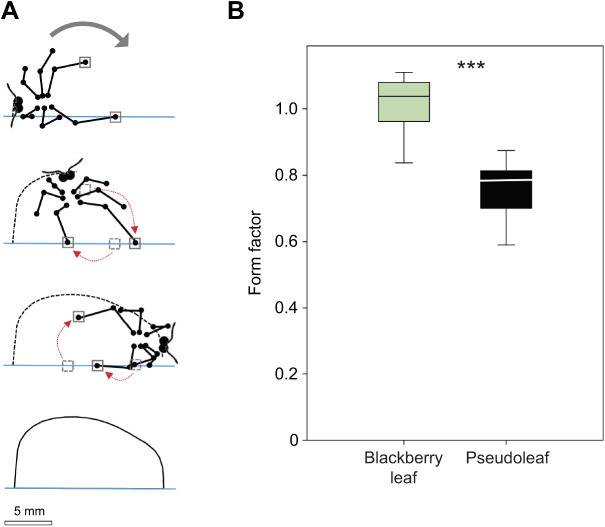
**Hind leg movements during the cutting trajectory and circularity of the fragments cut.** (A) Three drawings based on video images to illustrate marked changes in the leg positions and the anchoring points of the hind legs (grey squares) during cutting, as indicated by the red arrows. The fourth drawing shows the final fragment shape. (B) Comparison of the shape of fragments cut out of natural thin blackberry leaves and thin ^®^Parafilm pseudoleaves, using the form factor (for details, see Materials and Methods). *N*=17 blackberry leaves, *N*=27 pseudoleaves. Box plot shows median (line), 25–75 percentiles (box) and minimum–maximum values (whiskers); data are presented without outliers. ***P<0.001.

## RESULTS

### Do ants cut semicircular fragments?

Observations of the ants' leg movements during cutting of a pseudoleaf indicated that the hind leg position on both the leaf edge and lamina changed over the cutting trajectory (Fig. 3A) as the worker rotated. Foragers were never observed to displace themselves sidewards along the leaf edge while cutting, which would have led to the cutting of very large fragments even without significant changes in reach. The resulting cuts rarely resembled semicircles, as expected based on former descriptions of the cutting method of anchoring to the leaf edge and pivoting. Most fragments showed an initial cutting path perpendicular to the leaf edge, followed by a short but highly curved path and a subsequent straight path until the ant's body axis was perpendicular to the edge. Cutting then continued with lower curvature in the final half (Fig. 3A). Therefore, the curvature of most cutting paths was not exactly semicircular and showed some slight changes over the entire cut. Curvature was calculated as the reciprocal of the radius of curvature, every millimetre, from the hypothetical centre of curvature that corresponded to the middle point of the uncut fragment edge (Fig. S2). Regarding fragment shape, those cut out of soft blackberry leaves, with a very low leaf area density (0.18 mg mm^−2^), in the range of the softest leaves naturally harvested (Wetterer, 1991), had a form factor value around 1, i.e. they were approximately semicircular. Fragments cut out of thin pseudoleaves (area density of 0.14 mg mm^−2^) were semi-elliptical, with significantly lower values of the form factor, around 0.8 (Fig. 3B; Mann–Whitney *U*-test, *U*=12.0, *P*<0.001). Hence, ants changed the position of each hind leg as the anchoring point during their rotation and reduced their reach while cutting thin pseudoleaves, as compared with natural thin leaves.

### Effect of leaf thickness: flexibility in cutting behaviour

**Fig. 4.**
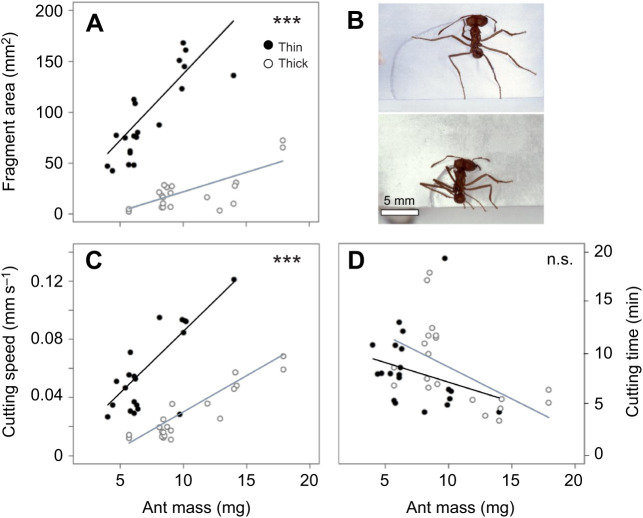
**Comparison of cutting behaviour in polymorphic ants cutting either thick or thin pseudoleaves.** (A) Influence of ant mass and pseudoleaf thickness on fragment size. Thin leaves, black circles, *N*=20; thick leaves, white circles, *N*=20; GLM, adjusted *R*²=0.85 (for detailed statistics, see Table S2A). (B) Pictures of foragers of comparable size cutting either a thin (top) or a thick (bottom) pseudoleaf. Photo credit: H. Heilmann. (C) Influence of ant mass and pseudoleaf thickness on cutting speed (adjusted *R*²=0.75; for detailed statistics, see Table S2B). (D) Influence of ant mass and pseudoleaf thickness on cutting time (adjusted *R*²=0.12; for detailed statistics, see Table S2C). ***P<0.001, n.s., not statistically significant; each symbol refers to the overall significance probability of the statistical model.

To investigate how leaf thickness influenced fragment size, cutting speed and cutting time, comparisons were made between fragments cut out of thin (*N*=20) and thick (*N*=20) pseudoleaves. Both ant mass and the thickness of the pseudoleaf had an effect on leaf fragment size (Fig. 4A; GLM, ant mass *P*<0.0001, pseudoleaf thickness *P*<0.0001; detailed statistics are given in Table S2A). In addition, there was an interaction effect between ant mass and pseudoleaf thickness, i.e. larger workers cut relatively larger fragments out of thin leaves as compared with thick leaves, a pattern that may reflect cutting with entirely extended legs by those workers that have the relatively longest hind legs. For most ants tested, fragment sizes were smaller when cut out of thicker material, resulting from an evident shortening in the extension of the legs anchoring to the leaf edge (Fig. 4B). Reduction of fragment area may occur to avoid overloading as would be expected if fragments cut from thick pseudoleaves were of similar area to those of thin pseudoleaves and therefore heavier, as a result of their higher area density. However, fragments cut from thick pseudoleaves were significantly lighter (Fig. S3; for statistics, see Table S1C), which indicates that the observed reduction in fragment size is not aimed at controlling the final mass of the harvested fragments. Cutting speed was also influenced by worker body mass (Fig. 4C; GLM, ant mass, *P*<0.0001; for detailed statistics, see Table S2B), with heavier ants cutting faster than lighter ants. Pseudoleaf thickness also affected cutting speed, with ants of similar size, in almost all cases, cutting slower when harvesting thick leaves. Cutting time, i.e. how long it took for an ant to completely cut a fragment, was not significantly influenced by either ant mass or leaf thickness (Fig. 4D; GLM, ant mass, *P*=0.47, leaf thickness, *P*=0.1; Table S2C).

Did ants, anchoring to the leaf edge while cutting, cut the largest possible fragments out of thin pseudoleaves? That is, how does the cutting behaviour on thin pseudoleaves compare with that on natural thin leaves? Ants were offered either thin pseudoleaves or thin blackberry leaves of very low leaf area density, i.e. 0.18 mg mm^−2^. The cutting material had an effect on the size of the resulting fragments (Fig. S4, GLM, *P*=0.049, Table S3A), as well as on cutting speed (GLM, *P*<0.0001, Table S3B) and cutting time (GLM, *P*<0.0001, Table S3C). There was, however, an interaction effect between ant mass and the cutting material for cutting speed, i.e. larger workers cut blackberry leaves at a relatively much higher speed than thin pseudoleaves. For most ants over the entire size range, workers cut slightly larger fragments, cut faster and spent a shorter total time while cutting blackberry leaves than while cutting thin pseudoleaves. Ant mass affected fragment area (GLM, *P*<0.001), but not cutting speed (GLM, *P*=0.42) and cutting time (GLM, *P*=0.9). Regarding fragment size determination, it is therefore argued that workers cut the softest natural leaves with their largest possible reach, i.e. larger fragments than this could not be cut without losing complete contact with the leaf edge. While cutting thin pseudoleaves, ants slightly reduced their maximal reach.

### Reach while cutting, with and without sensory feedback from the leaf edge

#### Reach of the inside hind leg while cutting

**Fig. 5.**
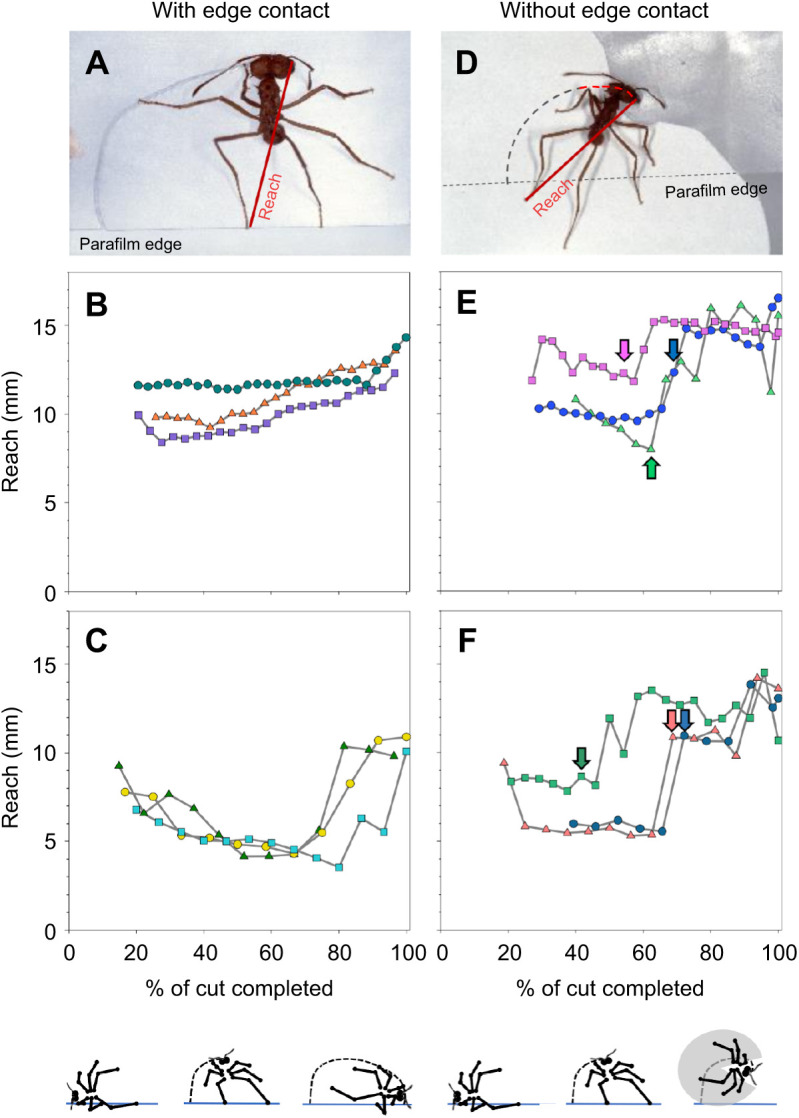
**Examples of the reach of the inside hind legs during the cutting trajectory, with and without contact with the leaf edge during the last half of the cutting trajectory.** (A) A control, non-manipulated worker cutting a thin pseudoleaf. (B,C) Three examples of reach for ants of comparable size (∼10 mg) while cutting either a thin (B) or a thick (C) pseudoleaf, with leg contact with the leaf edge. (D) A worker cutting a thin pseudoleaf after a piece of paper was inserted to prevent leg contact with the leaf edge. (E,F) Reach for three ants of comparable size (∼10 mg) while cutting a thin (E) or thick (F) pseudoleaf, without leg contact with the leaf edge. Arrows indicate the start of manipulation. Body and leg positions during a cut are schematically illustrated at the bottom. Photo credit: H. Heilmann.

As mentioned above, cutting ants modified the position of their hind legs on both the leaf edge and lamina during a cut, which could affect their reach. We first analysed reach of the inside hind leg, as workers use this leg to anchor themselves to the edge at the beginning of the cut. When anchoring to the edge of a thin pseudoleaf (Fig. 5A), the reach of the inside hind leg varied between 9 and 12 mm for ants of comparable size and did not change over the entire cut despite changes in leg position (Fig. 5B, three examples from ants with a body mass of ∼10 mg). When anchoring to the edge of thick leaves, reach was visibly reduced (Fig. 5C), and slightly decreased until almost the end, when a large increase occurred. This resulted from the ant drawing all its legs closer to its body while turning, and only suddenly extending the legs towards the end of the cut. In comparison, when leg contact with the leaf edge was prevented via manipulation with a paper sheet in the second half of the cut (Fig. 5D), workers slightly increased their reach after the manipulation when cutting thin leaves (Fig. 5E, arrows, *N*=14 total experiments performed), and greatly increase it when cutting thick leaves (Fig. 5F, arrows, *N*=10 total experiments performed). How these changes in hind leg extension correlate with differences in fragment-size determination is addressed below.

#### Reach of the outside hind leg while cutting

**Fig. 6.**
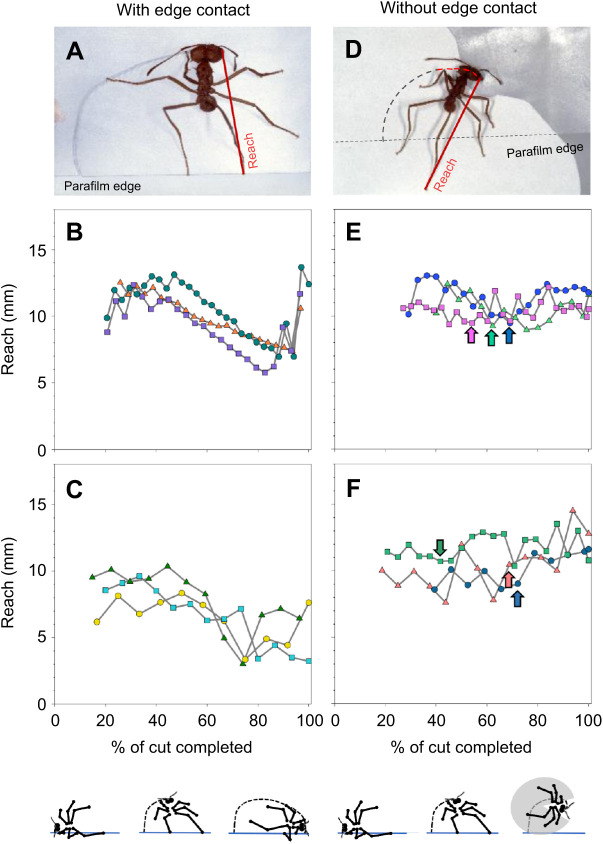
**Examples of the reach of the outside hind legs during the cutting trajectory, with and without contact with the leaf edge during the last half of the cutting trajectory.** (A) A control worker. (B,C) Three examples of reach for ants of comparable size (the same ants as in Fig. 5) while cutting either a thin (B) or a thick (C) pseudoleaf, with leg contact with the leaf edge. (D) A worker cutting a fragment after a piece of paper was inserted, preventing leg contact with the leaf edge. (E,F) Reach for three ants (the same ants as in Fig. 5) while cutting either a thin (E) or a thick (F) pseudoleaf without leg contact with the leaf edge. Body and leg positions during a cut are schematically illustrated at the bottom. Photo credit: H. Heilmann.

The outside hind leg anchors to the leaf edge in the second phase of a cut, after the ant's body axis has reached an approximately perpendicular position to the edge (Fig. 3A). In this phase, the inside hind leg usually moves away from the leaf edge, probably as a result of body rotation. The outside leg reach differed from that of the inside hind leg. Until the outside hind leg anchored to the leaf edge, at approximately 50% of the total cut, its reach was slightly variable but at comparable levels to those of the inside hind leg (Fig. 6B, same three ants as in Fig. 5). After anchoring to the edge, the reach steadily decreased because of leg flexion until the workers' body axis was parallel to the leaf edge, when reach strongly increased as workers performed the final rotation to sever the fragment. An approximately similar pattern occurred when ants cut thick pseudoleaves (Fig. 6C). When leg contact with the edge was prevented (Fig. 6D), the reach no longer steadily decreased but showed similar levels to those in the first half of the cut (Fig. 6E, arrows indicate the beginning of the manipulation). When ants cut thick leaves, they reacted to the manipulation by increasing their reach slightly (Fig. 6F). Figs 5 and 6 provide three detailed examples of reach from similarly sized ants over their cutting trajectory. A statistical analysis of average reach values is presented in the next section.

### Effect of loss of contact with the leaf edge on hind leg extension

**Fig. 7.**
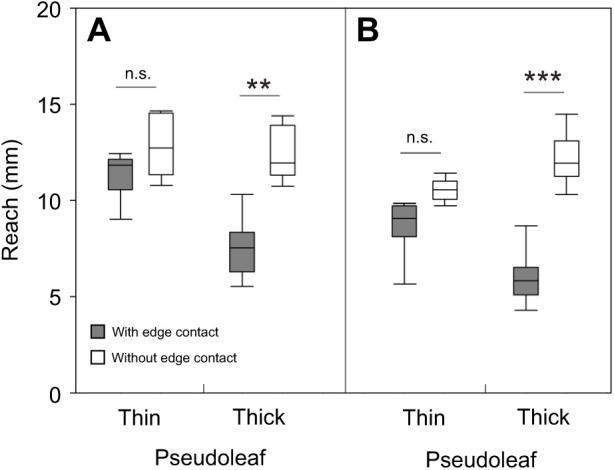
**Reach as determined by the inside and outside hind legs.** (A) Reach of the inside hind legs and (B) reach of the outside hind legs in control ants, i.e. ants having leg contact with the leaf edge, and in ants after their leg contact was prevented, for both thin (*N*=7 with contact, *N*=7 without contact) and thick (*N*=15 with contact, *N*=9 without contact) pseudoleaves. Measurements only correspond to the second half of the cutting trajectory for both groups. Range of worker body mass: 8–15 mg. Box plot shows median (line), 25–75 percentiles (box) and minimum–maximum values (whiskers); data are presented without outliers. **P<0.01, ***P>0.001; n.s., not statistically significant.

Fig. 7 summarises the reach of both inside and outside hind legs in workers ranging from 8 to 15 mg in body mass, for both non-manipulated ants, i.e. with leg contact with the leaf edge, and manipulated ants, i.e. without leg contact in the second half of their cut (thin: *N*=7 with contact, *N*=7 without contact, thick: *N*=15 with contact, *N*=9 without contact). To allow comparison, data from both groups correspond to values measured only during the second half of the cut. For the inside hind leg, losing contact with the leaf edge led to a slight but not statistically significant increase in workers’ reach when cutting thin pseudoleaves, yet to quite a significant increase when cutting a thick pseudoleaf, i.e. 5–10 mm before, compared with 10–15 mm after loss of contact (Fig. 7A, thin: Mann–Whitney rank sum test, *T*=50.0, *P*>0.8; thick: Mann–Whitney rank sum test, *T*=168, *P*<0.01). The same pattern could be observed for the outside hind leg, although workers stretched out this leg even more after losing contact with the leaf edge (Fig. 7B; thin: *t*-test, *t*=−0.94, *P*>0.4; thick: Mann–Whitney rank sum test, *T*=173, *P*<0.001). This indicates that as soon as the manipulated ants lost contact with the edge, they extended their hind legs and did not maintain the initial, reduced reach, probably for stability.

### Fragment size when sensory feedback from the leaf edge and from neck mechanosensory hairs is prevented

**Fig. 8.**
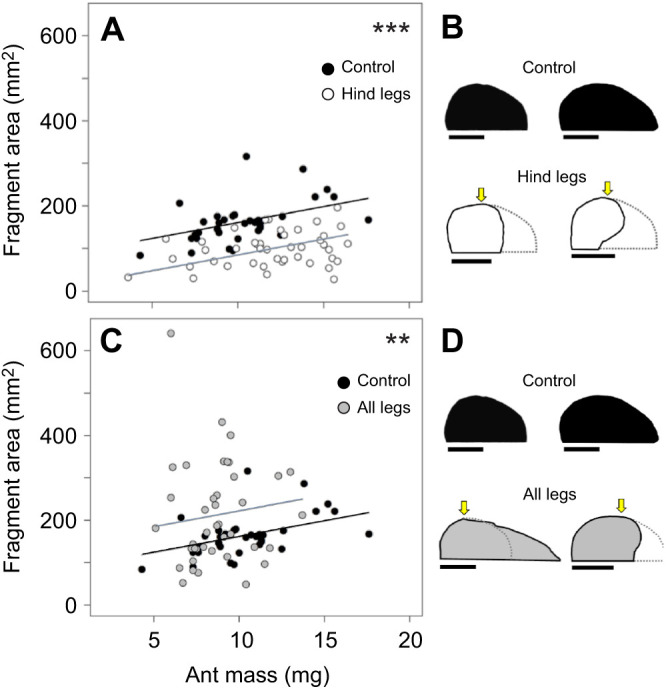
**Testing the hypothesis that contact with the leaf edge is one of the mechanisms underlying the determination of fragment size.** (A) Comparisons of the area of control fragments cut without any manipulation (*N*=35) and of fragments cut by ants without hind leg contact with the leaf edge (*N*=44). GLM with Tukey *post hoc* test, adjusted *R*²=0.31 (for detailed statistical analysis, see Table S4A,B). (B) Two examples of fragment shapes for the two series; the yellow arrows mark the beginning of each manipulation. Scale bars: 1 cm. (C) Areas of control fragments (data from A; *N*=35) and of fragments cut without contact of all legs with the edge (*N*=40). GLM with Tukey *post hoc* test, adjusted *R*²=0.31. (D) Two examples of fragment shapes for the two series. ***P<0.001, **P<0.01, each symbol refers to the overall significance probability of the statistical model.

If ants use reach while anchoring to the leaf edge to control cutting trajectory, ants precluded from contacting the edge with their hind legs, yet still able to anchor with their middle legs, should cut smaller fragments as compared with non-manipulated ants. Similarly, ants precluded from contacting the edge with all legs should be unable to control their cutting trajectory, unless an alternative control mechanism is involved. We compared the fragments cut by non-manipulated ants (*N*=35), ants with only their hind legs prevented from contact with the edge (*N*=44) and ants that lack contact with all legs (*N*=40). Leaf fragment area was influenced by both ant mass, as expected, and the manipulations, i.e. by the lack of contact with the edge with either their hind legs or all legs (Fig. 8A,C; GLM, ant mass, *P*<0.001; hind legs, *P*<0.0001; all legs, *P*<0.001; for detailed statistics, see Table S4A). Ants of a given size prevented from anchoring only their hind legs in the second half of the cut, but able to anchor their middle legs once they came into contact with the edge during their rotation, cut smaller fragments than those cut by non-manipulated workers (Fig. 8A; Tukey *post hoc* test, *P*<0.001; for detailed statistics, see Table S4B). Lack of sensory information about the leaf edge from all legs also influenced fragment size (Fig. 8C; Tukey *post hoc* test, *P*<0.01; Table S4B) and resulted in more variable fragment sizes compared with control fragments, suggesting the involvement of additional sensory information for the control of the trajectory. Interestingly, while some ants in the ‘all legs’ group cut approximately straight after the manipulation began, resulting in extremely large fragment sizes, others strongly rotated their body axis, therefore cutting roughly semi-elliptical fragments of smaller size (Fig. 8D, ‘all legs’ condition; see also Movie 2). Fragment sizes also differed significantly for ants cutting without hind leg contact with the edge and those cutting without contact of all six legs (Fig. 8A,C; Tukey *post hoc* test, *P*<0.001; Table S4B). Taken together, these results demonstrate the involvement of sensory information from contact with the leaf edge for control of the cutting trajectory. However, an additional feedback mechanism underlying the determination of fragment size should be involved, as half of the fragments cut without edge contact were similar in size to control fragments.

**Fig. 9.**
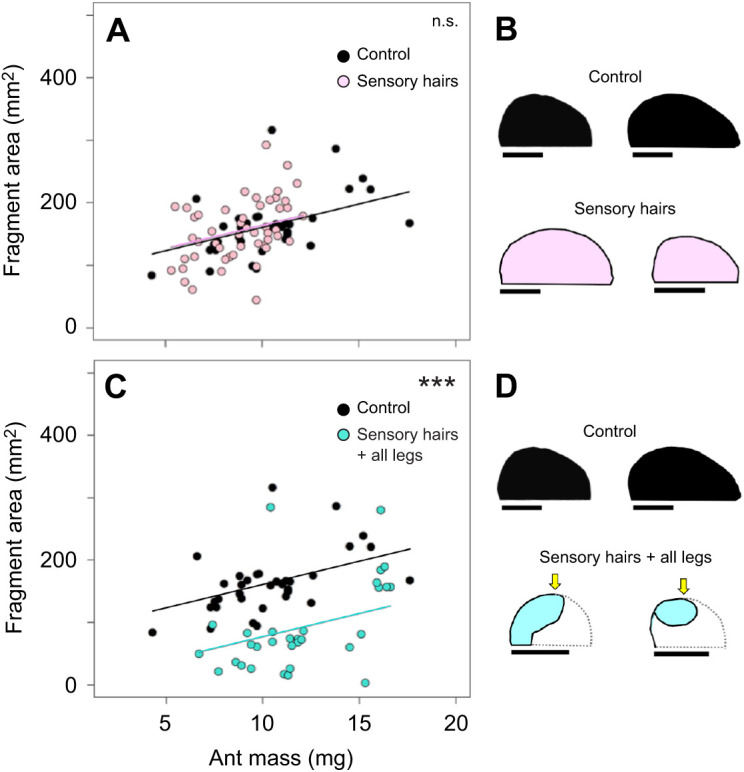
**Testing the hypothesis that sensory feedback of head movement is one of the mechanisms underlying the determination of fragment size.** (A) Comparison of the area of control fragments cut without any manipulation (*N*=35) and fragments cut by ants with ablated mechanosensory hairs on the neck joint, yet with leg contact with the leaf edge (*N*=50). GLM with Tukey *post hoc* test, adjusted *R*²=0.31 (for detailed statistics, see Table S4A,B). (B) Two examples of fragment shapes for the two series. Scale bars: 1 cm. (C) Area of control fragments (data from A; *N*=35) and of fragments cut by ants without feedback from both mechanosensory hairs and contact with the edge (*N*=31). GLM with Tukey *post hoc* test, adjusted *R*²=0.31. (D) Two examples of fragment shapes for the two series; yellow arrows mark the beginning of each manipulation. ***P<0.001; n.s., not statistically significant, each symbol refers to the overall significance probability of the statistical model.

To explore the involvement of an additional mechanism, we precluded information from head movements by ablating mechanosensory hair fields of the neck joint. Fig. 9 presents a comparison of fragment sizes between the three series: control (*N*=35), workers with ablated sensory hairs (*N*=50) and workers with ablated hairs and preclusion of sensory information from all legs regarding edge contact (*N*=31). While the removal of sensory hairs did not affect fragment area, the combination of hair ablation and lack of contact with the edge for all legs did (GLM, hairs, *P*=0.8; hairs and all legs, *P*<0.001; for detailed statistics, see Table S4A). Pairwise comparisons indicated that fragment sizes cut by workers with only ablated hair fields did not differ from those cut by non-manipulated workers (Fig. 9A; Tukey *post hoc* test, *P*=0.99; Table S4B). Fragment shapes also resembled those cut by non-manipulated workers (Fig. 9B). Fragments cut by non-manipulated workers differed in size from those cut by ants with ablated hairs and no feedback regarding leaf edge position from all legs (Fig. 9C; Tukey *post hoc* test, *P*<0.001); for ants of a given size, most manipulated workers cut smaller fragments than non-manipulated workers. In addition, fragments cut by workers with only ablated hairs differed from those cut by workers with ablated hairs and no leg contact with the edge (compare Fig. 9A and C; Tukey *post hoc* test, *P*<0.001). Many fragments of ants cutting with both ablated hair fields and no leg contact with the edge were very small and atypical in shape. They rather resembled small circles or ellipses (Fig. 9D, ‘sensory hairs+all legs’), resulting from a strong or complete rotation of the ant while cutting after losing contact with the edge in the second half of their cuts. Such atypical shapes were observed in 27% of the investigated ants in the series, compared with less than 5%, or none, in the other series.

Taken together, results indicate the existence of at least two mechanisms underlying the control of the cutting trajectory and the resulting fragment size. First, the use of sensory feedback from the leg contact with the leaf edge, which seems to be dominant. Second, the use of feedback from the bending of the head. Such sensory information seems not to be used to control the trajectory when workers could keep contact with the leaf edge, as observed in ants with ablated neck hairs only. However, the information could be used to control the cutting trajectory when leg contact with the edge is impeded or disarranged; for instance, when ants have to anchor on irregular leaf margins created by neighbouring cuts, and ant reach alone could not be reliably used to control the cutting path.

## DISCUSSION

### Plasticity of leaf cutting

Our study demonstrates that load size determination in leaf-cutting ants is plastic and does not simply result from a stereotypical geometric mode related to the worker's body size, as indicated in the literature (Lutz, 1929; Weber, 1972). Leaf-cutting ants have been shown to use flexible cutting rules to reduce the size of their harvested fragments; for instance, at the beginning of their foraging process, at shorter distances from the nest and as a response to increased leaf toughness (reviewed in Roces and Bollazzi, 2009). Based on the method of anchoring to the leaf edge while cutting, ants may simply shorten their reach to cut smaller fragments, but the maximal fragment size appears to be constrained by the maximum reach of an ant while rotating around its anchoring points. Interestingly, the largest fragment masses were only up to twice the cutting workers' body mass. However, leaf-cutting ants are able to carry much heavier loads, up to more than 6 times heavier than their body mass (Rudolph and Loudon, 1986), probably because fragments were disproportionate in shape as a result of irregular margins from neighbouring cuts.

So, why have leaf-cutting ants evolved a method of leaf cutting that apparently limits their resource intake per foraging trip, even though they are able to carry heavier loads? The answer may lie in the kinetics of load transport. Ants grasp fragments with their mandibles and carry them in an approximately upright position. Grass-cutting ants are able to adjust the position of the centre of mass of long grass fragments at the expense of a reduced walking speed, probably to avoid toppling (Moll et al., 2013). The roughly semicircular fragments of leaf-cutting ants are not elongated like grass blades, and it is therefore an open question why leaf-cutting ants do not cut even larger fragments. It has been suggested that most fragments carried by *Atta cephalotes* foragers, in the range of 2–3 times the workers' body mass, lie within the range that maximises the delivery rate of plant tissue (Rudolph and Loudon, 1986). However, the fragment sizes needed to maximise resource acquisition should be greater than the average fragment masses actually cut by foragers (Burd, 1996a,b; Segre and Taylor, 2019). Why do workers not cut larger fragments, considering that they would be able to carry them and thereby achieve higher delivery rates? Cutting behaviour is indeed flexible: foragers are able to reduce their reach while cutting smaller fragments out of thicker leaves. However, extending the reach while cutting beyond the values observed for thin leaves appears unfeasible, as suggested by our experimental manipulations. In evolutionary terms, any selection for the harvesting of even larger fragments should be linked with an increased reach while cutting because of the method of anchoring to the leaf edge, either with an enlargement of the hind legs or with a completely different cutting method. Interestingly, medium-sized ant foragers, as compared with minor and larger workers, have the relatively longest hind legs (Wetterer, 1991) and also perform most of the leaf-cutting, while minor and major workers perform other tasks. Leaf-cutting ants, in addition, tend to have relatively longer hind legs than grass-cutting ants (Fowler et al., 1986), which use a completely different method for cutting grass blades. This suggests that selection has in fact favoured relatively longer legs for ants rotating around pivots for cutting. Selection for larger fragments may also have been constrained by both handling requirements and the biomechanics of carriage: larger fragments may be energetically more costly to transport (Moll et al., 2012), and are more susceptible to influence by wind during carriage (Alma et al., 2016).

It is an open question why thick pseudoleaves were cut at a lower speed. Material thickness may influence the mandibles' opening angle, reducing the length of a single bite and increasing the number of total bites to sever a fragment. In addition, thicker material would probably require a higher force to be cut. The pseudoleaves used in our experiments had physical features comparable to those of natural leaves. Thin pseudoleaves had a lamina thickness of 0.13 mm, in the range of thin leaves, whereas the thick pseudoleaves were in the upper limit of thickness (0.38 mm) for tropical leaves (Kitajima and Poorter, 2010). Thin and thick pseudoleaves were cut at similar speeds to natural soft and tough leaves, respectively (Nichols-Orians and Schultz, 1989). However, characterising the cutting material by a single physical attribute does not accurately explain the ants' ability to cut it. For instance, natural blackberry leaves had a comparable low area density to thin ^®^Parafilm pseudoleaves, yet leaf-cutting ants cut blackberry leaves at a much higher speed. Importantly, leaf area density conflates leaf thickness and leaf mechanical properties, and it remains an open question which leaf properties ants respond to.

Interestingly, while leaf fragment size and cutting speed were both reduced when cutting thicker material, the total cutting time did not change as compared with thinner material. It is possible that ants use an estimate of the time actually spent during cutting and, depending on leaf thickness, appropriately adjust the cutting path to finish the cut after a fixed time. Nectar-feeding ants use estimates of time to make foraging decisions (Schilman and Roces, 2003). Controlling the time spent cutting may also be adaptive to reduce predatory and parasite risks while foraging (Orr, 1992). Alternatively, as cutting is an energetically extremely costly process (Roces and Lighton, 1995), ants could have a limited amount of energy immediately available for cutting a fragment. Conceivably, ants may adjust their cutting trajectory to the actual energy spent per unit length of the cut, irrespective of any time estimate, to not surpass a hypothetical maximal energy expenditure per fragment cut, an open question for future research.

### Contact with the leaf edge provides sensory information for the control of fragment size

The use of sensory information from contact with the leaf edge appears to be the main mechanism underlying fragment size determination as the ant anchors itself to the leaf edge with its hind legs and pivots around these points of contact. Fragment-size determination is not a simple function of body geometry, i.e. reach. Rather, workers reduce their reach when cutting thicker material by drawing their legs closer to their body, while keeping anchored to the leaf edge. Additionally, reach is not an invariant property even for a single fragment. Workers can also slightly displace the anchor of their hind legs, thus changing the pivot points (Fig. 3A), as also reported in *A. cephalotes* (van Breda and Stradling, 1994). The observed reach of the inside hind leg was rather stable throughout a cut, despite the pivot point moving slightly. This would lead to cutting with a certain radius at the beginning of a cut. Close to the point when the ant has turned to a perpendicular position to the leaf edge, the leading, outside hind leg also comes into contact with the leaf edge, while the inside leg usually releases contact. Once the contact of the outside leg is used as the second pivot point, the reach of this leg decreases steadily, as the ant rotates towards the point of contact. The use of that reach to control the cutting path could explain the visible changes in the trajectory in the second half of the cut.

The pattern described above was disrupted in our manipulations of all six legs, when sensory information from the location of the leaf edge was prevented in the last half of the cut. Without this feedback information, workers' reach did not keep steady, which would have resulted in the expected semi-elliptical fragments correlating in size with their body size. Rather, workers extended their legs more, increasing reach, which was especially noticeable when cutting thicker material without edge contact. As workers typically have contact with the leaf edge, they might use the contact as a point of reference to sense leg extension. The observed extension of their hind legs while cutting when this reference point was lacking could indicate that this posture is mechanically more suitable for applying the necessary forces to cut through the material.

In several workers cutting without edge contact, control over the cutting trajectory was noticeably impaired. Leg extension overall increased slightly after the legs lost contact with the edge, yet some fragments were smaller and round, because workers started to turn immediately. This observation highlights the importance of feedback of leaf edge position to control the cutting trajectory and to finish the cut. The movements of the leading outside legs during rotation may help the ant to explore the near environment on the leaf and to sense the location of the edge for the final trajectory of the cut. Active exploration before making a step or crossing a gap was observed in stick insects, cockroaches, crickets, and also foraging army ants plugging gaps (Dürr et al., 2018; Powell and Franks, 2007; Tuthill and Wilson, 2016), i.e. insects can actively adjust their leg movements after having gathered sensory information from leading legs. In addition, antennal exploration of the near environment to gather tactile information has been reported (Blaesing and Cruse, 2004; Schütz and Dürr, 2011), usually followed by leg movements. In leaf-cutting ants, we also observed that the antenna on the leading, outside side of the cutting ant moved quite frequently, stretching out and touching the leaf during cutting. At the end of a cut, when the ants got close to the leaf edge, the leading antenna was observed laterally touching the edge (example 1 in Movie 1). Guidance for the cutting path by information provided by antennal tactile information could be an additional mechanism to help determine the cutting trajectory.

When sensory information from the contact of the hind legs with the leaf edge is lacking, other legs anchoring to the edge can provide feedback. In our manipulations only on the hind legs, the reduced reach being attained with the middle legs led to the cutting of smaller fragments. A similar effect was described in the garden spider *Araneus diadematus*, which uses its leg reach as a reference during web building. The use of regenerated, shorter legs leads to a reduction in spacing of the capture spiral of their webs (Vollrath, 1987).

Unexpected changes in cutting direction can also occur when other legs get into contact with additional leaf margins caused by neighbouring cuts from other ants. Deviations from an expected semi-elliptical shape do not necessarily lead to a reduction in fragment size. In Movie 3, the outside, middle leg of a forager accidentally contacted the perpendicular edge of the offered pseudoleaf, while both hind legs were still anchored to the initial edge. As a reaction to this new mechanosensory input, the ant changed its cutting trajectory towards this margin, resulting in an unexpectedly larger fragment despite a shorter entire cutting length.

### Control of head position as a feedback mechanism for fragment-size determination

Even without feedback from the location of the leaf edge, most ants were still able to cut roughly semi-elliptical fragments of the same or slightly larger size as compared with fragments cut with feedback from the leaf edge. This indicates the existence of another mechanism aiding the determination of leaf fragment size. While van Breda and Stradling (1994) could not find a relationship between the ants' reach and the cutting arc, they reported that a tighter curve, resulting from a strong bending of the head, was seen as soon as the cutting trajectory approached the thicker leaf veins of natural leaves. Our observations on ants cutting a combined pseudoleaf with a thin margin and a thicker lamina (Fig. 1) clearly indicated that workers actively controlled the cutting trajectory irrespective of mechanical changes in the cutting material. They did not keep their expected, concave cutting trajectory in the thin, easy-to-cut material, but bent the head in the final part to perform an unexpected convex trajectory and finally harvest a larger fragment.

Ablating the neck's mechanosensory hair fields probably led to the lack of neuronal signalling originating from the bending of such hairs, as observed when ablating proprioceptive hair fields on the antennae of stick insects (Jaske et al., 2021; Theunissen et al., 2014). We observed loss of control over the cutting trajectory only when feedback from mechanosensitive hairs was prevented in combination with the lack of sensory information provided by the legs' contact with the leaf edge. The resulting strong rotation while cutting might have resulted from a pronounced bending of the head to get sensory feedback for its position, and the concomitant adjustment of the body position. This suggests the existence of at least two mechanisms underlying the determination of leaf fragment size: as long as ants have contact with the leaf edge, sensory feedback from the leaf edge is used to control the cutting trajectory, irrespective of the sensory information provided by head movements. When workers have no contact with the leaf edge, they can continue to keep control over their cutting trajectory by using information provided by the neck's mechanoreceptors. When information from both feedback mechanisms is completely excluded, workers lose control over the cutting trajectory.

### Conclusions

Based on our findings, we propose that ants do not use a single fixed reach to determine the size of the fragments cut, but display a plastic mode of cutting that relies on sensory information provided by at least two different feedback loops. Feedback from the limbs, predominantly the hind legs, appears to be the main mechanism providing directional information of the location of the leaf edge, while the subordinate guidance of the cutting trajectory by feedback from the head's movements exerts control over the cutting trajectory to a lesser extent.

## Supplementary Material

10.1242/jexbio.244246_sup1 Supplementary information
