# Supplementary material for: Two feedback mechanisms involved in the control of leaf fragment size in leaf-cutting ants
Source: J Exp Biol. 2023 Jun 23;226(12):jeb244246. doi: 10.1242/jeb.244246 (PMC10323230; doi:10.1242/jeb.244246)
Supplement: Supplementary information [file jexbio-226-244246-s1.pdf]

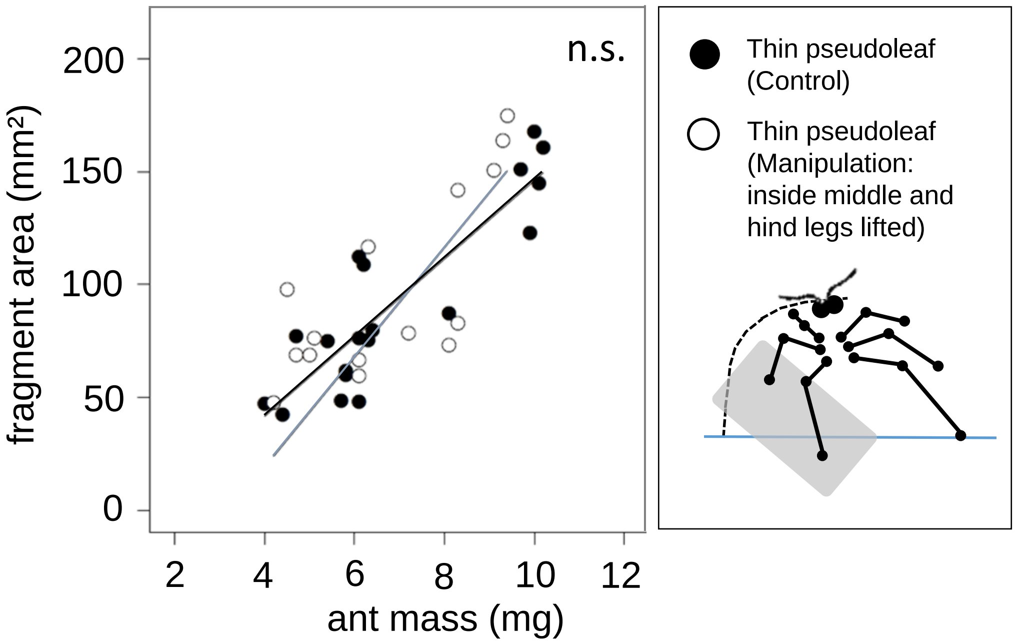


**Fig. S1. Experiment to demonstrate that manipulations using a paper sheet to lift legs of an ant while cutting do not lead to disturbances that influence the cutting behaviour**. Relationship between ant mass and fragment mass for control, unmanipulated ants cutting thin ®Parafilm pseudoleaves (black circles, $N_{control}$=20) and for manipulated ants cutting the same material, the inside middle and hind legs of which were lifted (white circles, $N_{manipulation}$=15). For more details of the experimental manipulations see main text. Groups do not differ statistically, GLM, Adjusted $R^2$ = 0.83, Tukey post-hoc test P=0.7 (For detailed statistical analysis, see Tables S1A and S1B further below). The significance symbol refers to the overall significance probability of the statistical model.

Journal of Experimental Biology • Supplementary information

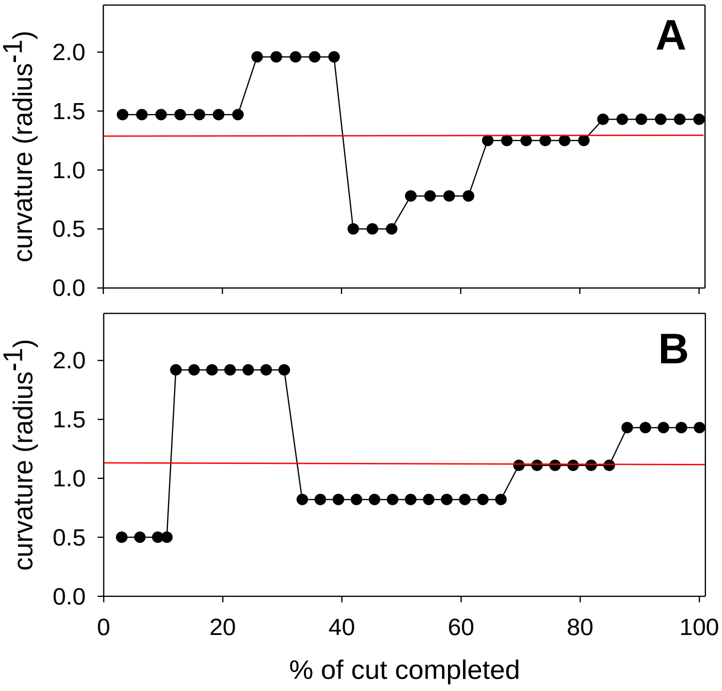


**Fig. S2. Workers do not cut a fragment by maintaining an invariant curvature over the entire cutting path.** Shown are two examples of changing curvatures of the cutting path for two different ants of similar body mass. (A) ant mass 10.1 mg, (B) ant mass 10 mg. Cutting material: thin pseudoleaf. Each point marks 1 mm of the cutting trajectory (cutting length). The horizontal lines describe the calculated mean curvature of the ant's cutting trajectory. Curvature was calculated as the reciprocal of the radius of curvature, every mm, from the hypothetical centre of curvature that corresponded to the middle point of the uncut fragment edge.

Journal of Experimental Biology • Supplementary information

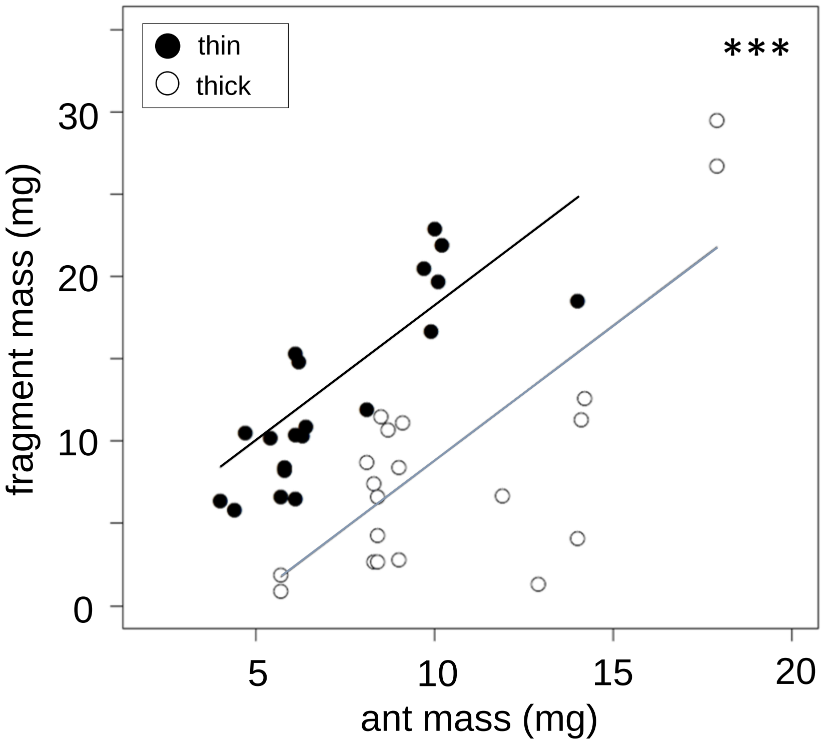


**Fig. S3. Ants foraging on thick pseudoleaves cut fragments of reduced area as compared to those cut out thin pseudoleaves (see main text). Hypothetically, such reduction in fragment area may occur to avoid the overloading that would be expected if fragments cut from thick pseudoleaves were of similar area that those of thin pseudoleaves. However, fragments from thick pseudoleaves were significantly lighter than those of thin pseudoleaves as shown in this figure, i.e., the observed reduction in fragment size for thick leaves is not aimed at controlling the mass of the harvested fragments.** Depicted are the relationships between ant mass and fragment mass for ants cutting either thin or thick ®Parafilm pseudoleaves. $N_{thin}$=20, $N_{thick}$=20. GLM, adjusted $R^2$ = 0.61. Influence on fragment mass: ant mass (centered), P<0.001; leaf (Parafilm) thickness, P<0.001 (For detailed statistical analysis, see Table S3 further below). The significance symbol refers to the overall significance probability of the statistical model.

Journal of Experimental Biology • Supplementary information

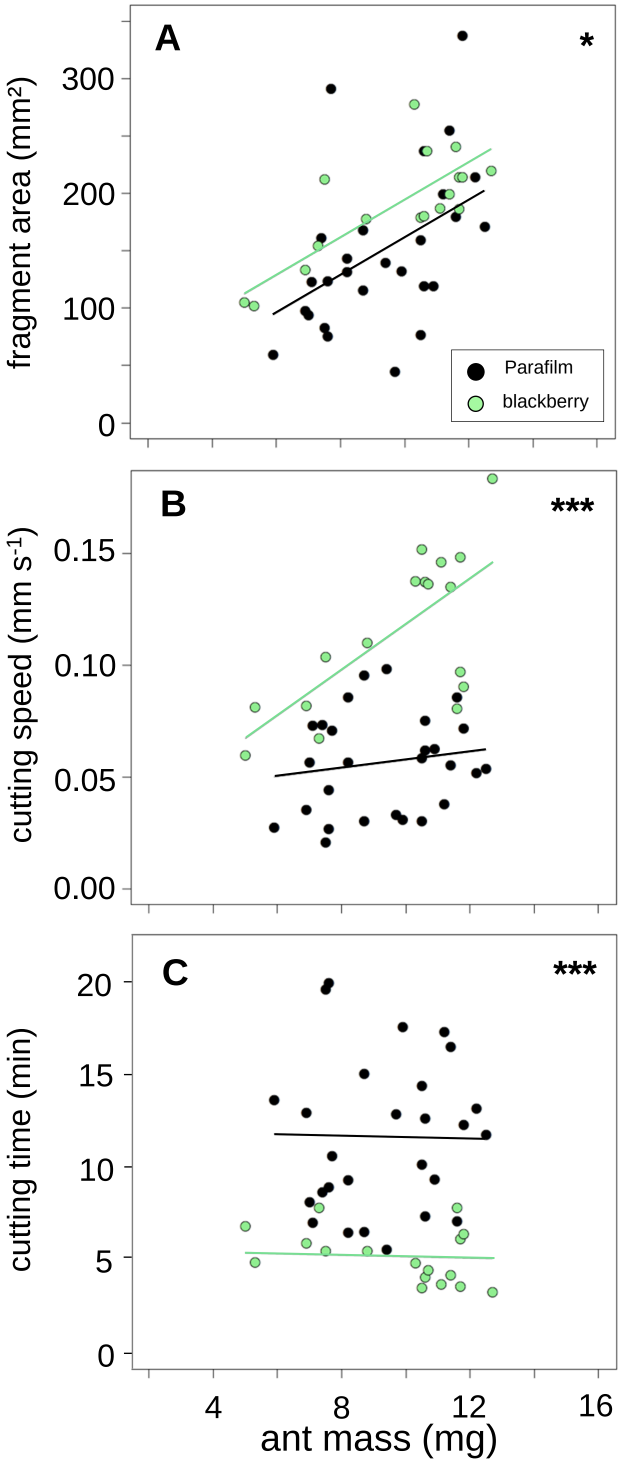


Journal of Experimental Biology • Supplementary information

**Fig. S4. Comparisons between ants cutting ®Parafilm pseudoleaves and natural blackberry leaves.** Compared are: (A) fragment area, (B) cutting speed, and (C) cutting time between fragments cut either from thin ®Parafilm pseudoleaves (mean area density = 0.14 mg*(mm$^2$)$^{-1}$) or from natural blackberry leaves, *Prunus fruticosus* (mean area density = 0.18 mg*(mm$^2$)$^{-1}$). GLM, fragment area, adjusted $R^2$ = 0.34, $P<0.05$, cutting speed, $R^2$ = 0.6, $P<0.001$, cutting time, $R^2$ = 0.45, $P<0.001$ (For detailed statistical analysis, see Table S6). The significance symbols within each plot frame refer to the overall significance probability of the statistical model.

Journal of Experimental Biology • Supplementary information

**Table S1. R statistics** (A) GLM for Figure S1 (supplementary), fragment area; the group „Parafilm thick“ is not shown in the figure, but was included in the model to be consistent with the analysis; *formula = y ~ antmass_centered * parafilm* (B) Tukey post-hoc test for Figure S1 (supplementary) and Figure 4A (main text), *formula = y ~ antmass_centered * parafilm* (C) GLM for Figure S3 (supplementary), fragment mass, *formula = y ~ antmass_centered + parafilm*

| | Estimate | Standard Error | t value | Pr(>\|t\|) | |
|---|---|---|---|---|---|
| **A** | | | | | |
| (Intercept) | 116.147 | 5.6 | 20.738 | < 2e-16 | *** |
| Ant mass centered | 17.428 | 2.287 | 7.620 | 8.31e-10 | *** |
| Parafilm thick | -103.339 | 7.703 | -13.414 | < 2e-16 | *** |
| Parafilm two legs | 6.896 | 8.787 | 0.785 | 0.436 | n.s. |
| Ant mass centered : parafilm thick | -13.588 | 2.626 | -5.175 | 4.43e-06 | *** |
| Ant mass centered : parafilm two legs | -0.719 | 3.69 | -0.195 | 0.846 | n.s. |
| **B** | | | | | |
| Thin : thick | -103.339 | 7.704 | -13.414 | < 1e-05 | *** |
| Two legs : thick | 110.234 | 8.593 | 12.829 | < 1e-05 | *** |
| Two legs : thin | 6.896 | 8.787 | 0.785 | 0.714 | n.s. |
| **C** | | | | | |
| (Intercept) | 14.505 | 1.003 | 14.459 | < 2e-16 | *** |
| Ant mass centered | 1.642 | 0.227 | 7.237 | 1.37e-08 | *** |
| Parafilm thick | -9.437 | 1.557 | -6.062 | 5.17e-07 | *** |

Journal of Experimental Biology • Supplementary information

**Table S2.** R statistics, GLM (A) Figure 4A (main text), fragment area, *formula = y ~ antmass_centered * parafilm,* (B) Figure 4C (main text), cutting speed, *formula = y ~ antmass_centered * parafilm*, (C) Figure 4D (main text), cutting time, *formula = y ~ antmass_centered * parafilm*

| | Estimate | Standard Error | t value | Pr(>\|t\|) | |
|---|---|---|---|---|---|
| **A** | | | | | |
| (Intercept) | 107.746 | 4.617 | 23.337 | 2e-16 | *** |
| Ant mass centered | 13.134 | 1.705 | 7.704 | 4.0e-09 | *** |
| Parafilm thick | -94.937 | 6.816 | -13.928 | 4.56e-16 | *** |
| Ant mass centered : parafilm thick | -9.294 | 2.098 | -4.431 | 8.42e-05 | *** |
| **B** | | | | | |
| (Intercept) | 0.067 | 0.003 | 19.756 | < 2e-16 | *** |
| Ant mass centered | 0.009 | 0.001 | 6.927 | 4.1e-08 | *** |
| Parafilm thick | -0.049 | 0.005 | -9.713 | 1.34e-11 | *** |
| Ant mass centered : parafilm thick | -0.004 | 0.002 | -2.626 | 0.013 | * |
| **C** | | | | | |
| (Intercept) | 482.27 | 52.25 | 9.229 | 5.06e-11 | *** |
| Ant mass centered | -20.13 | 19.3 | -1.043 | 0.304 | n.s. |
| Parafilm thick | 124.16 | 77.15 | 1.609 | 0.116 | n.s. |
| Ant mass centered : parafilm thick | -17.38 | 23.74 | -0.732 | 0.469 | n.s. |

Journal of Experimental Biology • Supplementary information

**Table S3.** R statistics, GLM for Figure S4 (supplementary) with blackberry leaves (A) fragment area, *formula = y ~ antmass_centered + treatment* (B) cutting speed, *formula = y ~ antmass_centered * treatment* (C) cutting time, *formula = y ~ antmass_centered + treatment*

| | Estimate | Standard Error | t value | Pr(>\|t\|) | |
|---|---|---|---|---|---|
| **A** | | | | | |
| (Intercept) | 152.543 | 10.098 | 15.107 | < 2e-16 | *** |
| Ant mass centered | 16.404 | 3.833 | 4.279 | 0.00011 | *** |
| Treatment blackberry | 32.979 | 16.288 | 2.025 | 0.049 | * |
| **B** | | | | | |
| (Intercept) | 0.056 | 0.005 | 12.034 | 7.16e-15 | *** |
| Ant mass centered | 0.002 | 0.002 | 0.820 | 0.417 | n.s. |
| Treatment blackberry | 0.056 | 0.007 | 7.512 | 3.66e-09 | *** |
| Ant mass centered: treatment blackberry | 0.008 | 0.004 | 2.235 | 0.0311 | * |
| **C** | | | | | |
| (Intercept) | 699.931 | 39.488 | 17.725 | <2e-16 | *** |
| Ant mass centered | -2.361 | 14.991 | -0.158 | 0.876 | n.s. |
| Treatment blackberry | -385.891 | 63.695 | -6.058 | 3.57e-07 | *** |

Journal of Experimental Biology • Supplementary information

**Table S4.** R statistics, Figure 8 ad 9 (main text) (A) GLM, *formula = y ~ antmass_centered + treatment* (B) Tukey post-hoc test, *formula = y ~ antmass_centered + treatment*

| | Estimate | Standard Error | t value | Pr(>\|t\|) | |
|---|---|---|---|---|---|
| **A** | | | | | |
| (Intercept) | 162.596 | 11.688 | 13.911 | < 2e-16 | *** |
| Ant mass centered | 7.475 | 1.940 | 3.852 | 0.0002 | *** |
| Manipulation all legs | 61.065 | 16.190 | 3.772 | 0.0002 | *** |
| Manipulation hind leg | -76.147 | 15.993 | -4.761 | 3.74e-06 | *** |
| Manipulation hairs | 3.125 | 15.469 | 0.202 | 0.84 | n.s. |
| Manipulation hairs + legs | -83.887 | 17.374 | -4.828 | 2.77e-06 | *** |
| **B** | | | | | |
| All legs : hairs + legs | -144.952 | 17.620 | -8.226 | < 1e-04 | *** |
| Control : all legs | 61.065 | 16.190 | 3.772 | 0.002 | ** |
| Hairs : all legs | -57.939 | 14.573 | -3.976 | < 0.001 | *** |
| Hind legs : all legs | -137.211 | 16.247 | -8.445 | < 1e-04 | *** |
| Control : hairs + legs | -83.887 | 17.374 | -4.828 | < 1e-04 | *** |
| Hairs : hairs + legs | 87.013 | 16.897 | 5.15 | < 1e-04 | *** |
| Hind legs : hairs + legs | 7.741 | 16.215 | 0.477 | 0.989 | n.s. |
| Hairs : control | 3.125 | 15.469 | 0.202 | 0.99 | n.s. |
| Hind legs : control | -76.147 | 15.993 | -4.761 | < 1e-04 | *** |
| Hind legs : hairs | -79.272 | 15.461 | -5.127 | < 1e-04 | *** |

**Table S5. Raw data for Figs 3–9 and Figs S1–S4.**

Click here to download Table S5

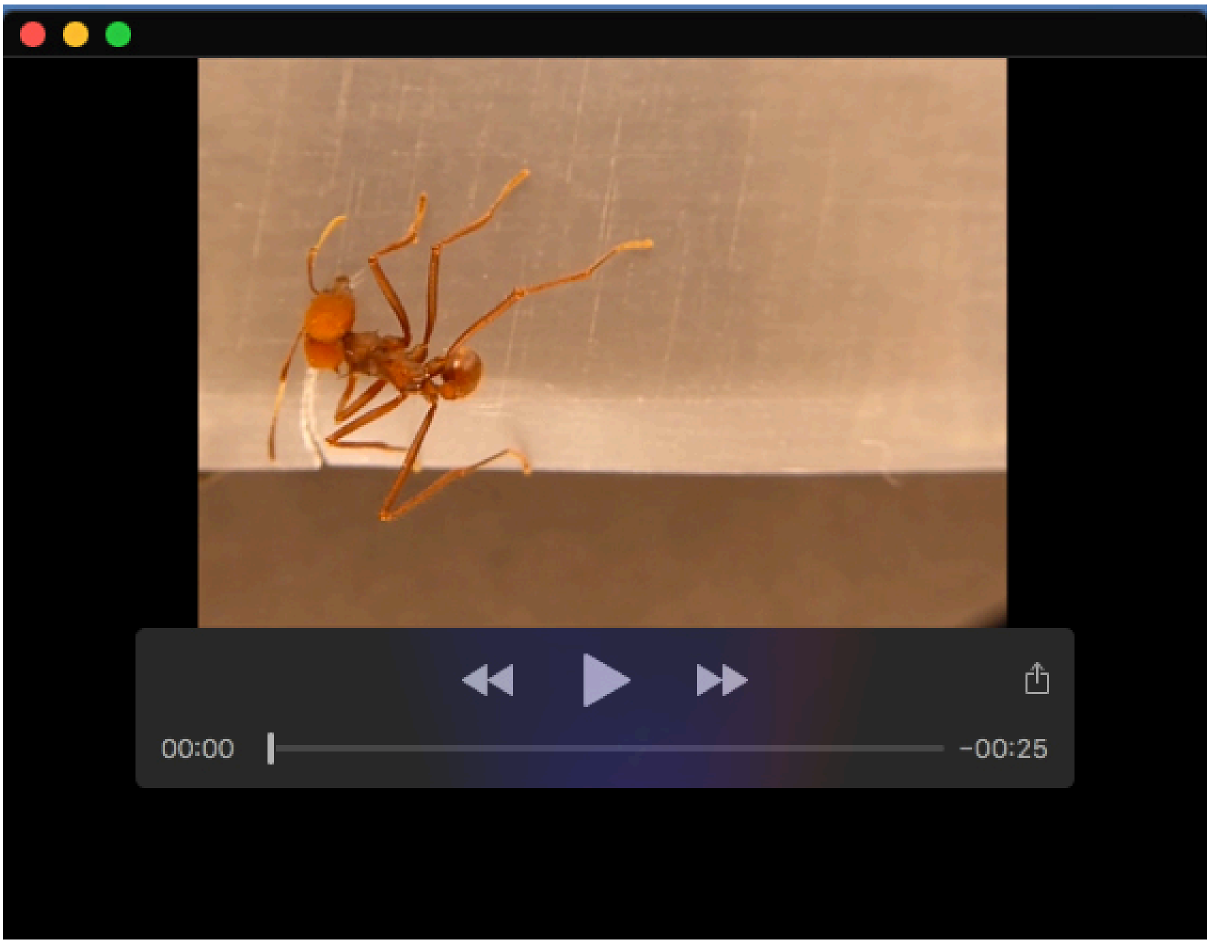


**Movie 1.** Two examples of workers cutting a fragment from a thin ®Parafilm pseudoleaf. Example 1: usual cutting behaviour with a leaf edge - leaf edge 180° rotation; example 2: manipulation experiment, in which a sheet of paper was introduced between the workers and the pseudoleaf to prevent leg contact with the leaf (The shown cuts were not included in our data analysis, because of the special conditions of illumination for the videos).

Journal of Experimental Biology • Supplementary information

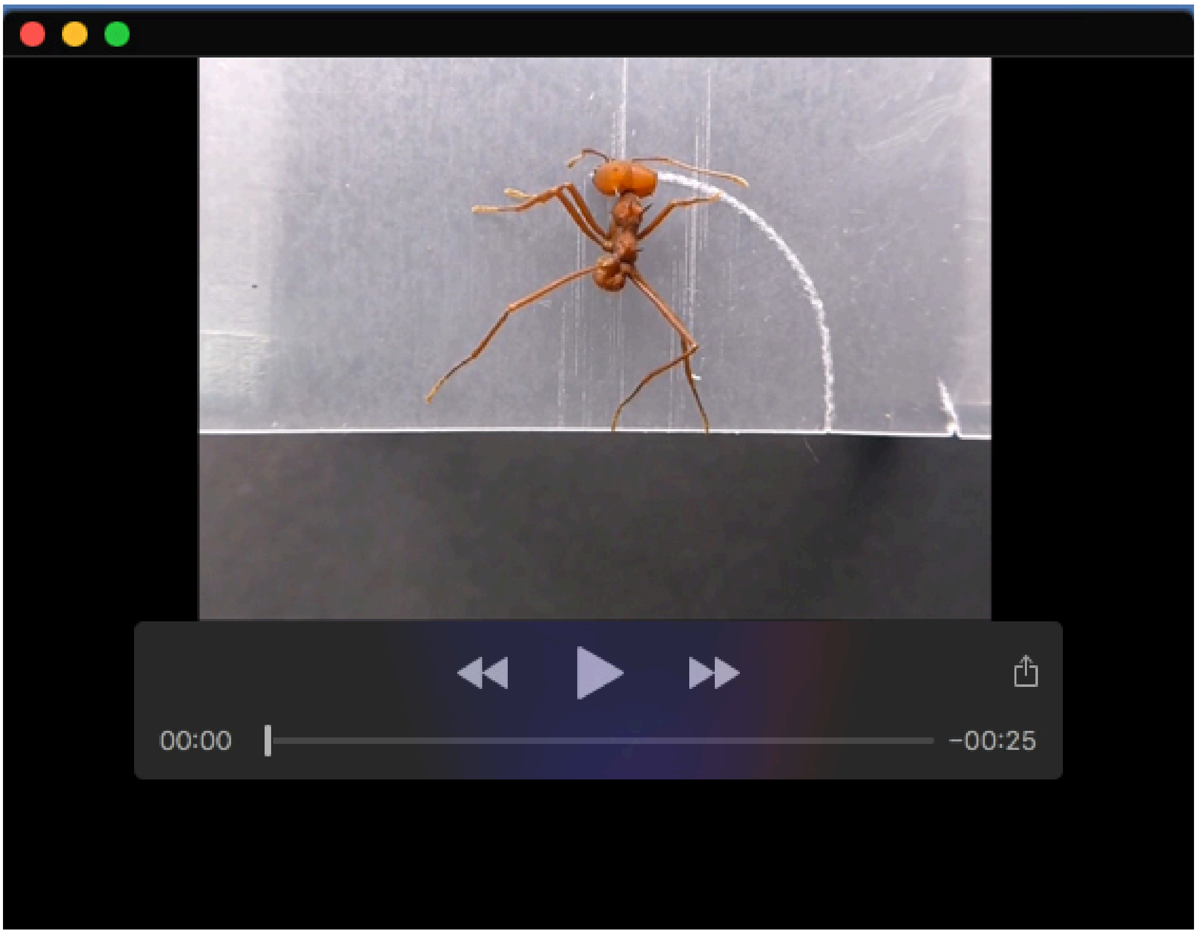


**Movie 2.** An additional example cutting a thin ®Parafilm pseudoleaf using a paper sheet to prevent contact of the legs with the leaf edge, in which the ant strongly rotated its body during the experimental manipulation.

Journal of Experimental Biology • Supplementary information

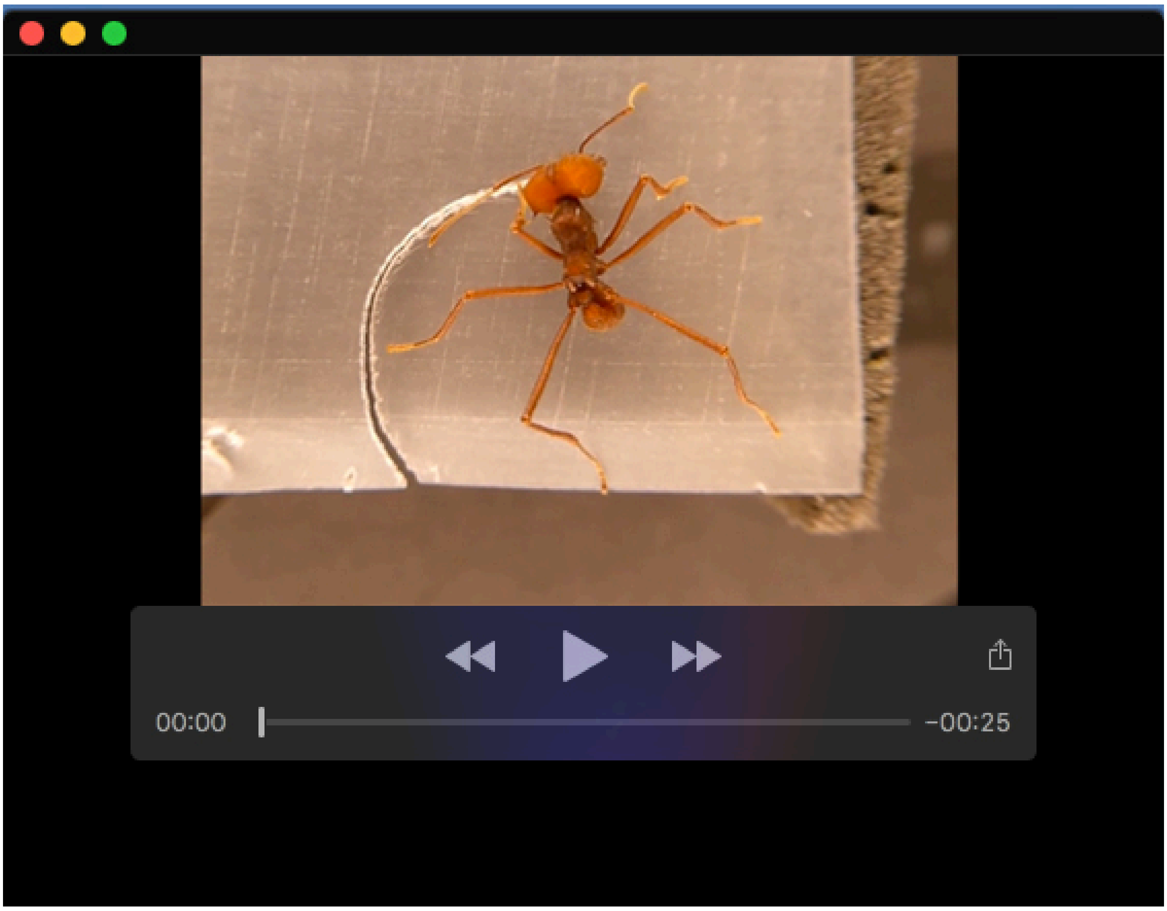


**Movie 3.** An example cutting a thin ®Parafilm pseudoleaf showing behavioural flexibility and the relevance of sensory information obtained from the position of the leaf edge to control the shape of the fragment to be cut: after contact with the perpendicular leaf edge, the ant changed the direction of the cut and continued along a straight line.
